# Latent Squint

**Published:** 1896-06-06

**Authors:** Robert Brudenell Carter

**Affiliations:** Consulting Ophthalmic Surgeon to St. George's Hospital


### Medical Progress and Hospital Clinics.

[*The Editor will be glad to receive offers of co-operation and contributions from members of the profession. All letters should be addressed to* The Editor, at the Office, 428, Strand, London, W.C.]

### LATENT SQUINT.

By Robert Brudenell Carter, F.R.C.S., Consulting Ophthalmic Surgeon to St. George's Hospital.

(*Continued from page* 375.)

It is obvious that the occurrence of latent squint may depend either upon excessive action of some muscle or muscles, or upon defective action of the antagonist or antagonists; and that the question as to which of these conditions may be present will not always be a simple one. Let us take, for example, a case of right exophoria, in which when at rest, and not stimulated by the presence of a known object of vision, the right eye will be divergent relatively to the left. Let us assume, farther, that the symptoms and general condition justify the belief that the effort of overcoming this relative divergence during the performance of the visual act, an effort necessary for the maintenance of single vision, is a source of ocular strain and fatigue, occasioning headaches or other more or less troublesome and disabling conditions, and requiring to be relieved by art. Even in this, the simplest form of the affection, there will be some difficulty in ascertaining whether the relative divergence depends upon weakness of the internal rectus or upon over action of the external; and, until some conclusion upon this point has been arrived at, it will be difficult to determine whether tenotomy of the latter muscle, or advancement of the former, will be most likely to fulfil the objects of the operator. Even in simple exophoria, however, it by no means follows that the faulty position is due entirely to excess or defect on the part of one of the two muscles which are apparently at fault. It is an elementary proposition in mechanics that the path of a moving body is determined by the sum of all the forces which are brought to bear upon it. Taking the simplest illustration, if a ball is pulled by two forces acting at right angles to each other, it will move along the diagonal between them. In like manner, if the position of the eye is changed by deficiency or excess of force on the part of some single muscle, the ultimate resulting position will not be determined by this deficiency or excess alone, but by the readjustment of all the forces (or muscles) which are concerned in acting upon the organ. Forgetfulness of this fact has led to the exercise of much misapplied ingenuity in the construction of diagrams, intended to show, from the relative positions of the two images in diplopia, what muscle is in default as the cause of it.

In order to meet this difficulty, and to assist the surgeon in his choice of operative measures, Dr. Stevens, of New York, has recently devised an ingenious instrument, which he calls a "tropometer," or "measurer of turning." It consists of a chin and face rest, so arranged that the head of the patient may be steadily supported without being subjected to any irksome restraint. At the other end of the board which supports the rest there is fixed a sort of telescope, turned, for the sake of convenient management, so as to be at right angles to the patient's face, and having a prism in its object-glass so as to give a clear image of the eye under inspection to the observer. The telescope has a screw focal adjustment, an adjustment by which it can be raised or lowered, another by which it can be placed nearer to the eye or farther from it, and a sort of micrometer scale in its eyepiece, marked with two dark lines, and with finer lines between them, numbered in degrees. An external stud allows the micrometer scale to be turned a quarter of a circle, so as to render its lines either vertical or horizontal, the former position serving to measure the extent of lateral, the latter the extent of vertical rotation. The head of the patient being fixed, the telescope is so adjusted that an inverted image of the cornea of the eye under examination accurately fills the interval between the two dark lines of the micrometer scale, and the patient is then made to look in succession upwards, downwards, to the right and to the left, the movements of the margin of the cornea in each direction being read off upon the scale. It is necessary to guard carefully against unconscious movements of the head, which may be steadied by the examiner or by an assistant, and it is desirable to repeat every movement several times in each direction, and to secure that the largest ocular excursion within the powers of the patient is accomplished. After each movement the eye should return to its starting position, and the cornea should again accurately fill the space between the two dark lines. Dr. Stevens believes that a restricted range of movement may be accepted as evidence of the weakness of the muscle by which that movement is mainly accomplished, and that an unusually extensive range may be accepted as evidence of excessive action. The instrument is so new that I have not at present had sufficient opportunity of arriving at definite conclusions as to its practical value; but for this purpose I have arranged to institute a series of examinations upon a large number of patients with no ocular defects, in order to obtain something like a standard of the normal ranges of movement, and of the differences in this respect which may be displayed by different individuals, or by the two eyes of the same individual. I have, however, used the tropometer in some of the few cases of latent squint on which I have at present operated, and have been induced by its indications to select tenotomy in lieu of advancement; but in none of these has sufficient time elapsed to afford complete certainty of the permanence of the change for the better which in each of them has for the time being been produced. It must be remembered that troublesome consequences from heterophoria are chiefly met with in patients of highly neurotic temperament, since others would be likely to overcome the muscular difficulty without feeling any excessive strain; and hence that operations for the relief of ocular troubles of this kind are performed, almost exclusively, upon people in a condition at least approaching to morbidity, and apt to be greatly influenced, either for good or for evil, by anything which strongly impresses the imagination. Such people become the natural prey of quacks and impostors; and there is only too much reason to believe that operations for latent squint, in America if not in this country, have been performed in a large number of cases in which it would be difficult or impossible to justify them.

